# Pomaglumetad Methionil (LY2140023 Monohydrate) and Aripiprazole in Patients with Schizophrenia: A Phase 3, Multicenter, Double-Blind Comparison

**DOI:** 10.1155/2014/758212

**Published:** 2014-03-19

**Authors:** David H. Adams, Lu Zhang, Brian A. Millen, Bruce J. Kinon, Juan-Carlos Gomez

**Affiliations:** Lilly Research Laboratories, Eli Lilly and Company, Lilly Corporate Center, Indianapolis, IN 46285, USA

## Abstract

We tested the hypothesis that long-term treatment with pomaglumetad methionil would demonstrate significantly less weight gain than aripiprazole in patients with schizophrenia. In this 24-week, multicenter, randomized, double-blind, Phase 3 study, 678 schizophrenia patients were randomized to either pomaglumetad methionil (*n* = 516) or aripiprazole (*n* = 162). Treatment groups were also compared on efficacy and various safety measures, including serious adverse events (SAEs), discontinuation due to adverse events (AEs), treatment-emergent adverse events (TEAEs), extrapyramidal symptoms (EPS), and suicide-related thoughts and behaviors. The pomaglumetad methionil group showed significantly greater weight loss at Week 24 (Visit 12) compared with the aripiprazole group (−2.8 ± 0.4 versus 0.4 ± 0.6; *P* < 0.001). However, change in Positive and Negative Syndrome Scale (PANSS) total scores for aripiprazole was significantly greater than for pomaglumetad methionil (−15.58 ± 1.58 versus −12.03 ± 0.99; *P* = 0.045). The incidences of SAEs (8.2% versus 3.1%; *P* = 0.032) and discontinuation due to AEs (16.2% versus 8.7%; *P* = 0.020) were significantly higher for pomaglumetad methionil compared with aripiprazole. No statistically significant differences in the incidence of TEAEs, EPS, or suicidal ideation or behavior were noted between treatment groups. In conclusion, long-term treatment with pomaglumetad methionil resulted in significantly less weight gain than aripiprazole. This trial is registered with ClinicalTrials.gov NCT01328093.

## 1. Introduction

All of the current antipsychotics target dopamine receptors as their common mechanism of action [1, 2]. Due to differences in the affinities to the dopamine receptors and interactions with other biogenic monoamine receptors, therapeutic profiles and limitations of individual drugs vary [1]. The conventional/typical antipsychotic drugs are effective in treating positive symptoms of schizophrenia [3, 4]. However, they are associated with extrapyramidal symptoms (EPS) and hyperprolactinemia [3, 4]. The newer generation atypical antipsychotics not only improve positive and, to some extent, negative symptoms of schizophrenia [5, 6] but also have a lower propensity to cause EPS [3, 4, 7, 8] and hyperprolactinemia [3, 4]. However, these agents may still be associated with other adverse events (AEs), such as body weight gain, lipid abnormalities [9, 10], and glucose dysregulation [3, 4] in some patients. Furthermore, drug-induced weight gain may affect long-term compliance, which directly influences the likelihood of successfully managing the course of disease [11, 12]. Hence, a significant need exists to develop treatments for schizophrenia that are not associated with these and other AEs.

Pomaglumetad methionil (LY2140023 monohydrate), a methionine prodrug of the active compound LY404039, is a specific and potent metabotropic glutamate 2/3 (mGlu2/3) receptor agonist. It is devoid of affinity to all biogenic amine receptors, including dopamine receptors [1], but demonstrates a preclinical pharmacological profile similar to that of clinically effective atypical antipsychotic drugs [13, 14]. Unlike currently approved antipsychotic medications, pomaglumetad methionil was not expected to be associated with weight gain, EPS, or metabolic side effects because of its selective receptor binding profile [14] and hence has been suggested as an alternative treatment for schizophrenia [15]. Previous studies—a proof-of-concept study [15], a Phase 2, inpatient, dose-ranging study [13], a Phase 2 long-term safety study [16], and an acute, fixed-dose, Phase 2 study [17]—showed that treatment with pomaglumetad methionil was well-tolerated and demonstrated a low incidence of AEs, such as weight gain, EPS, and hyperprolactinemia, that are typically observed with currently available dopaminergic antipsychotic treatments. Efficacy results were as follows: positive in the proof-of-concept study [15], inconclusive in the Phase 2, inpatient, dose-ranging study, [13] and negative in the acute, fixed-dose, Phase 2 registration study [17].

Aripiprazole, a quinolinone derivative, is a novel atypical antipsychotic drug which has a mechanism of action distinct from other atypical antipsychotic drugs as it is a partial dopamine D_2_ receptor agonist with a lower association with weight gain [3]. Because pomaglumetad methionil also has a low likelihood of inducing weight gain and may even be associated with weight loss in patients previously treated with antipsychotics [16], aripiprazole was selected as an appropriate comparator in this weight gain study to demonstrate a potentially unique weight profile for pomaglumetad methionil.

The present study was part of a Phase 3 clinical development program for pomaglumetad methionil in the treatment of schizophrenia. The purpose of the present study was to compare the mean weight gain in flexibly dosed pomaglumetad methionil (20, 40, or 80 mg, twice daily [BID]) with flexibly dosed aripiprazole (10, 15, or 30 mg/day) in patients with schizophrenia after 24 weeks of double-blind treatment. The study was intended to help characterize the benefits and risks of pomaglumetad methionil for a broad population of patients with schizophrenia but was stopped early when the pomaglumetad methionil schizophrenia monotherapy development program was stopped, based on lack of efficacy in an acute placebo-controlled efficacy study [17] as well as early stopping of a second acute trial due to futility [18]. This early stopping had minimal impact on the double-blind active treatment phase results of the present study, however, since enrollment was complete and 97% of patients had already completed or discontinued the double-blind active treatment phase of the study at the time of the termination.

## 2. Materials and Methods

### 2.1. Participants

This study was conducted at 57 centers in 10 countries. Male and female outpatients, aged 18 to 65 years (inclusive) with a diagnosis of schizophrenia, as defined by the* Diagnostic and Statistical Manual of Mental Disorders, Fourth Edition, Text Revision* (DSM-IV-TR) [19] and confirmed by Structural Clinical Interview for DSM-IV-TR (SCID), were enrolled into the study. Patients were excluded for the following reasons: (1) if they had other current Axis I psychiatric diagnoses (as defined in DSM-IV-TR) in addition to schizophrenia, (2) if they had a history of inadequate clinical response, in the opinion of the investigator, to antipsychotic treatment for schizophrenia (inadequate clinical response for this study was defined as persistent and moderately severe hallucinations, delusions, or thought disorder after completion of 2 or more antipsychotic medication trials of at least 8 weeks duration in the past 12 months prior to Visit 1), (3) if they had aripiprazole treatment within the past 2 months, (4) if they had a DSM-IV-TR diagnosis of substance abuse or dependence, (5) if they had a substance-induced psychosis by DSM-IV-TR criteria, or (6) if they were pregnant or breast-feeding. Written informed consent was obtained from the participants at the screening visit.

### 2.2. Study Design

This was a multicenter, randomized, double-blind, Phase 3 study to assess the safety and efficacy of pomaglumetad methionil (flexibly dosed between 20 and 80 mg BID) in patients with schizophrenia. An active control, aripiprazole (flexibly dosed between 10 and 30 mg/day), was included for comparison.

This study was divided into 3 study periods. Study Period I was a screening and antipsychotic drug taper/discontinuation phase (3–10 days). During this period, all screening procedures were completed and previous antipsychotic medications were stopped. Study Period II was the double-blind active treatment phase. Patients who met enrollment criteria during Study Period I continued into Study Period II and were randomized in 3 : 1 ratio to either pomaglumetad methionil or aripiprazole. The duration of the double-blind active treatment phase (24 weeks) enabled assessment of the safety of pomaglumetad methionil over a longer period of time than acute efficacy studies. Patients who completed Study Period II continued into Study Period III, which was an open-label active treatment phase. The duration of the open-label active treatment phase (28 weeks) enabled collection of additional safety data.

### 2.3. Ethical Considerations

The study was conducted in accordance with consensus ethics principles derived from international ethics guidelines, including the Declaration of Helsinki and Council for International Organizations of Medical Sciences International Ethical Guidelines, International Conference on Harmonization Guideline for Good Clinical Practice E6, and applicable laws and regulations.

### 2.4. Safety Measures

Safety measures that were monitored at every visit included weight, AEs, vital signs, suicidality as measured by the Columbia Suicide Severity Rating Scale (C-SSRS) [20], results of a neurological examination, and waist circumference (except at screening). Weight was assessed using a calibrated digital scale and investigator sites were instructed to assess patient's weight at a consistent time of day in light consistent clothing and by the same person at each visit. All serious adverse events (SAEs) and treatment-emergent adverse events (TEAEs) were reported according to terminology in the Medical Dictionary for Regulatory Activities (MedDRA), version 15.1. Other safety parameters that were monitored throughout the study were electrocardiogram (ECG) intervals and laboratory analytes, as well as EPS and abnormal movements, which were evaluated using the Barnes Akathisia Scale (BAS) [21], the Simpson-Angus Scale (SAS) [22], and the Abnormal Involuntary Movement Scale (AIMS) [23].

### 2.5. Efficacy Measures

Efficacy scales which were assessed/collected throughout the study were the Positive and Negative Syndrome Scale (PANSS) [24], Clinical Global Impression-Severity (CGI-S) [25], and the 16-Item Negative Symptom Assessment (NSA-16) [26].

### 2.6. Statistical Analysis

An expected sample size of at least 450 patients in the pomaglumetad methionil group and 150 patients in the aripiprazole group provided at least an 88% chance to demonstrate that the pomaglumetad methionil group had about 1.8 kilograms (kg) less mean body weight increase from baseline compared with the aripiprazole group at a 1-sided 0.025 alpha level. Sample size was based on a 2-sample* t*-test, assuming that the standard deviation of body weight change from baseline would be approximately 6 kg. The sample ratio of 3 : 1 was chosen to maximize exposure to pomaglumetad methionil.

The analyses were conducted on the intent-to-treat (ITT) population that included all patients according to the treatment group to which they were assigned and received at least 1 dose. The ITT set was modified prior to data lock, excluding patients (*n* = 3) from a site with an International Conference on Harmonisation Good Clinical Practices noncompliance issue. Analyses of the double-blind active treatment phase included comparisons between treatment groups with 2-sided tests conducted at the 0.05 alpha level. Data collected during the open-label active treatment phase were summarized.

The primary objective was evaluated with a mixed-model repeated measures (MMRM) analysis. The model included the fixed categorical effects of treatment, gender, pooled site, visit, treatment-by-visit interaction, and prior olanzapine use (yes or no, where “yes” was defined as usage of olanzapine for >7 cumulative days during the 6 weeks prior to screen), as well as the continuous, fixed covariates of baseline and baseline-by-visit interaction. The within-patient errors were modeled according to an unstructured covariance matrix. The Kenward-Roger approximation was used to estimate denominator degrees of freedom. The primary contrast was the pomaglumetad methionil versus aripiprazole comparison at Week 24 (Visit 12). Based on the visitwise treatment contrasts from the primary analysis, a sequential testing procedure was used to determine (with appropriate type I error control) the earliest time point at which the treatments differed statistically in mean weight change. The incidence of patients meeting criteria for potentially clinically significant weight gain (i.e., ≥7% increase from baseline) or weight loss (i.e., ≥7% decrease from baseline) at endpoint or any time during the double-blind active treatment phase was compared between treatment groups using the Cochran-Mantel-Haenszel test [27], controlling for baseline body mass index (BMI) and prior olanzapine use.

Incidence rates of safety categorical variables were summarized and compared between treatment groups using Fisher's exact test. The change from baseline in vital signs, ECG, SAS total score, AIMS total score (items 1–7), and BAS global score was assessed using an MMRM analysis with a model similar to that used for the efficacy analysis. The change from baseline to the last observed measure in the laboratory analytes was ranktransformed prior to analysis and was assessed using an analysis of variance (ANOVA) model with treatment as a fixed effect.

All the efficacy variables were evaluated with MMRM analysis. The model included the fixed, categorical effects of treatment, gender, pooled site, visit, treatment-by-visit interaction, and predefined subpopulation (yes/no), as well as the continuous, fixed covariates of baseline and baseline-by-visit interaction.

Reason for discontinuation, baseline characteristics, baseline efficacy, and baseline EPS measures along with illness characteristics were summarized by treatment group. Continuous measures were analyzed with a single factor ANOVA model with fixed effect of treatment and Fisher's exact test was used to compare categorical data between treatment groups. Time-to-discontinuation (due to AEs, lack of efficacy, and for any reason) was analyzed separately with Kaplan-Meier estimated survival curves [28], and the log-rank test was used for comparisons. All statistical analyses were performed using SAS, version 9.2 (SAS Institute, Inc., Cary, NC, USA).

## 3. Results

### 3.1. Patient Characteristics and Disposition

**Figure 1:**
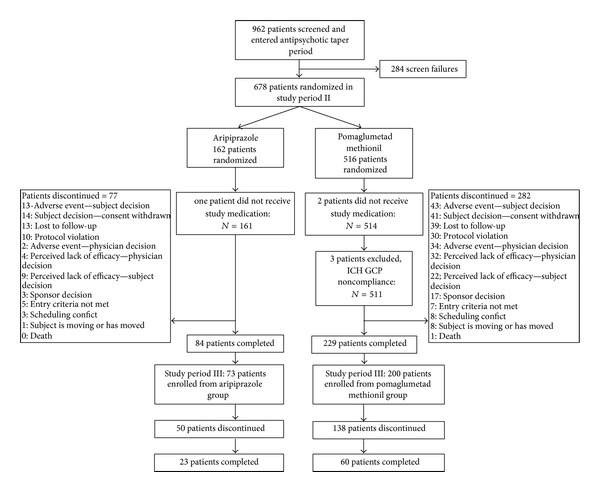
Patient disposition.

Of the 962 patients screened, 678 were randomized in an approximately 3 : 1 ratio (516 in the pomaglumetad methionil group and 162 in the aripiprazole group) during the double-blind active treatment phase, with 672 patients (511 in the pomaglumetad methionil group and 161 in the aripiprazole group) making up the ITT population (Figure 1). Baseline patient characteristics were comparable between the treatment groups (Table 1). Most of the patients were male (64.3%), white (52.4%), and from the United States (67.4%). The mean (standard deviation [SD]) for age was 42.45 (10.88) years, for baseline weight 89.90 (22.21) kg, for BMI 30.44 (7.39) kg/m^2^, and for waist circumference −101.11 (17.10) cm. The major reasons for discontinuation across treatment arms during the double-blind active treatment phase were as follows: AE-subject decision (8.3%), subject decision-consent withdrawn (8.2%), lost to follow-up (7.7%), protocol violation (6.0%), AE-physician decision (5.4%), perceived lack of efficacy-physician decision (5.4%), and perceived lack of efficacy-subject decision (4.6%). Reasons for discontinuation were comparable between pomaglumetad methionil and aripiprazole groups except for AE-physician decision (6.7% versus 1.2%, resp.; *P* = 0.005).

A total of 313 patients completed the double-blind active treatment phase (229 in pomaglumetad methionil arm and 84 in aripiprazole arm) from which 272 patients enrolled into the open-label active treatment phase. Of 83 patients who completed the open-label active treatment phase, 60 had taken pomaglumetad methionil during the double-blind active treatment phase. The most common reason for discontinuation during the open-label active treatment phase was sponsor decision (48.9%), reflecting early termination of the study following negative efficacy of an acute (Phase 2) registration trial [17], as well as early stopping of a second (Phase 3) registration trial due to futility [18].

**Figure 2:**
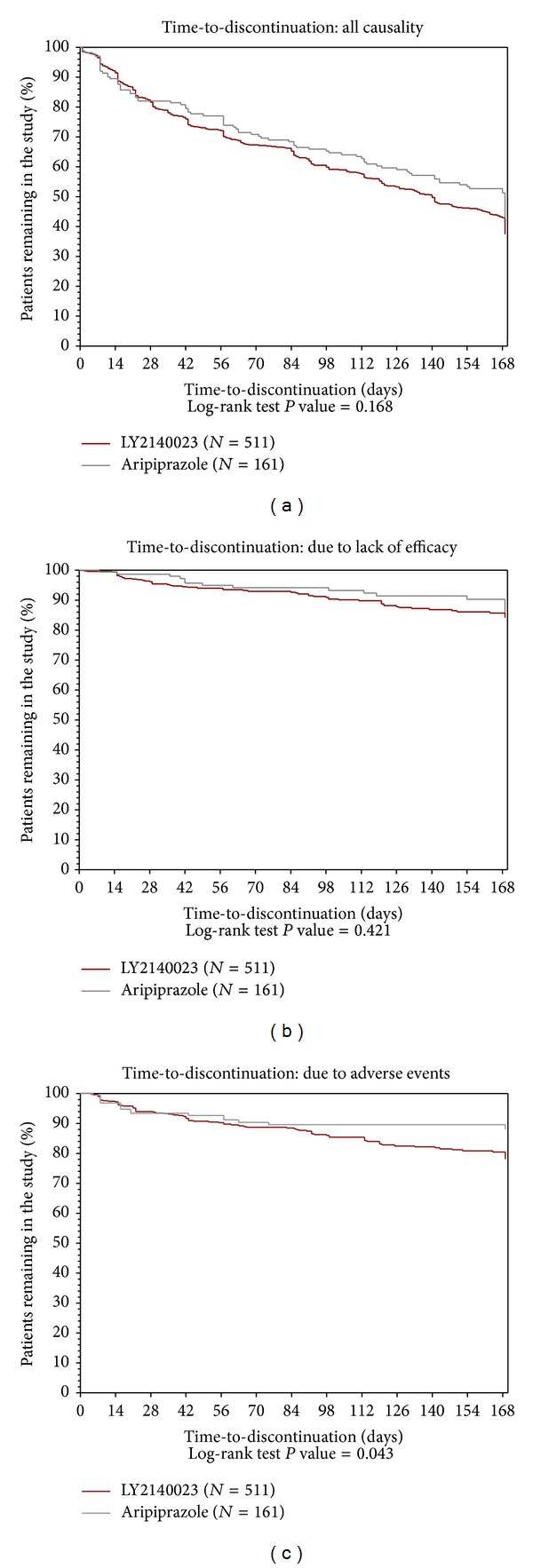
Kaplan-Meier plots showing time-to-discontinuation (all causality, due to lack of efficacy, and due to adverse events) for intent-to-treat (ITT) patients with schizophrenia in pomaglumetad methionil (*N* = 511) and aripiprazole (*N* = 161) treatment groups for 24 weeks of double-blind treatment.

There were no statistically significant differences between the pomaglumetad methionil and aripiprazole treatment groups in time-to-discontinuation (all causality) and time-to-discontinuation due to lack of efficacy. However, time-to-discontinuation due to AEs through the double-blind active treatment phase was significantly shorter for patients in the pomaglumetad methionil group than for those in the aripiprazole group (*P* = 0.043; Figure 2).

### 3.2. Safety Measures

#### 3.2.1. Body Weight

**Figure 3:**
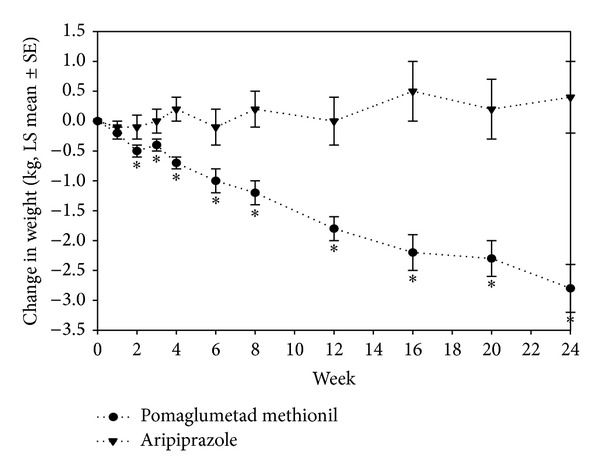
Least-squares mean change of the weight from mixed-effects model repeated measures among intent-to-treat (ITT) patients with schizophrenia in pomaglumetad methionil (*N* = 511) and aripiprazole (*N* = 161) treatment groups for 24 weeks of double-blind treatment. *P ≤ 0.05. *P* values are from type III tests of LS mean differences between treatments at each visit from MMRM. LS mean: least-squares means, MMRM: mixed-effects model with repeated measures, and SE: standard error.

A statistically significant difference was observed between pomaglumetad methionil and aripiprazole groups in least-squares (LS) mean change for weight at Week 24 (Visit 12 LS mean change [SE]: −2.8 [0.4] kg versus 0.4 [0.6] kg, resp.; *P* < 0.001). The onset of statistically different mean weight changes between pomaglumetad methionil and aripiprazole treatment group was at Week 2 (Visit 4 LS mean change [SE]: −0.5 [0.1] kg versus −0.1 [0.2] kg; *P* = 0.016; Figure 3). A significantly larger percentage of pomaglumetad methionil-treated than aripiprazole-treated patients reported ≥7% loss of baseline body weight at endpoint (13.1% versus 3.2%; *P* < 0.001) and at any time (15.6% versus 4.5%; *P* < 0.001) during the double-blind active treatment phase, while no significant findings were reported between pomaglumetad methionil-treated and aripiprazole-treated patients for ≥7% weight gain at endpoint (4.1% versus 7.1%, resp.) and at any time (5.1% versus 8.4%, resp.).

#### 3.2.2. Serious Adverse Events (SAEs)

In total, 47 of 672 (7.0%) patients experienced at least 1 SAE during the double-blind active treatment phase. There was a significant difference in the incidence of SAEs between pomaglumetad methionil-treated and aripiprazole-treated patients (8.2% versus 3.1%, resp.; *P* = 0.032; Table 2); however, no significant differences were reported between the treatment groups in the incidence of individual SAEs. The most common SAE among pomaglumetad methionil-treated and aripiprazole-treated patients was schizophrenia (2.9% versus 1.2%, resp.). One death (completed suicide) was reported in the pomaglumetad methionil treatment group but was judged by the investigator not to be treatment-related. During the open-label active treatment phase, 12 (4.4%) patients experienced at least 1 SAE.

#### 3.2.3. Discontinuations due to Adverse Events (AEs)

A total of 97 patients (14.4%) discontinued due to AEs, with significantly more patients discontinuing in the pomaglumetad methionil treatment group than in the aripiprazole treatment group due to AEs (16.2% versus 8.7%; *P* = 0.020; Table 2). The most common AE that resulted in discontinuation among both pomaglumetad methionil-treated and aripiprazole-treated patients was schizophrenia (2.9% and 1.2%, resp.). No significant difference was reported between the treatment groups in the incidence of any individual AEs that resulted in discontinuation. A total of 14 (5.2%) patients discontinued the open-label active treatment phase due to AEs.

#### 3.2.4. Treatment-Emergent Adverse Events (TEAEs)

A total of 479 (71.3%) patients experienced ≥1 TEAE during the double-blind active treatment phase. AEs reported with ≥3% incidence are shown in Table 3. Nausea, reported by 17.3% of patients, was the most frequent TEAE; however, only 1.3% of all patients discontinued the study because of nausea. Significantly more pomaglumetad methionil-treated patients reported nausea compared with aripiprazole-treated patients (19.2% versus 11.2%; *P* = 0.023). Significantly more aripiprazole-treated patients compared with pomaglumetad methionil-treated patients reported akathisia (7.5% versus 2.5%; *P* = 0.007), dyspepsia (3.7% versus 1.0%; *P* = 0.027), pyrexia (2.5% versus 0.4%; *P* = 0.032), and nasal congestion (1.9% versus 0.2%; *P* = 0.045). During the open-label active treatment phase, 114 (42.2%) patients experienced ≥1 TEAE (compared to the maximum severity observed during the double-blind phase). Headache (5.9%), nausea (5.6%), and insomnia (3.0%) were the most frequent TEAEs (≥3%) during the open-label phase.

#### 3.2.5. Extrapyramidal Symptoms (EPS)

Baseline EPS were comparable between the treatment groups (Table 4). No significant differences were found between treatment groups at the end of the double-blind active treatment phase. There were also no statistically significant differences based on categorical analysis of EPS scales (data not shown).

#### 3.2.6. Suicidality

There were no statistically significant differences between treatment groups in measures of suicidal ideation and suicidal behavior, as assessed with the CSSRS during the double-blind active treatment phase. Forty-six (9.4%) patients in the pomaglumetad methionil group and 7 (4.5%) patients in the aripiprazole group had treatment-emergent suicidal ideation (*P* = 0.064), and 7 (1.4%) patients in the pomaglumetad methionil group and no patients in the aripiprazole group had suicidal behavior (*P* = 0.205) compared to baseline.

#### 3.2.7. Additional Safety Measures

There were no significant differences between treatment groups in the incidence of treatment-emergent neurological exam findings, except abnormal gait, which was significantly higher in the aripiprazole treatment group than the pomaglumetad methionil group (2.5% versus 0.4%; *P* = 0.032) during the double-blind active treatment phase.

There were no clinically relevant laboratory findings for the pomaglumetad methionil group, and there were no clinically significant findings on vital signs or ECGs for the pomaglumetad methionil group compared with the aripiprazole group during the double-blind active treatment phase. Both treatment groups had significant within-group decreases in cholesterol and small but significant within-group increases in fasting glucose, but there were no significant differences between treatment groups. There was no significant change from baseline in triglycerides for either of the treatment groups. Based on the National Cholesterol Education Program (NCEP) [29] criteria, the percentage of patients exhibiting a shift from normal/borderline to high triglycerides was statistically significantly lower for the pomaglumetad methionil group than for the aripiprazole group at any time (11.6% versus 21.3%; *P* = 0.010) and at endpoint (4.5% versus 10.7%; *P* = 0.026). There were no other significant treatment differences in shifts in the NCEP criteria. There was a significantly greater mean decrease from baseline in prolactin for the pomaglumetad methionil group compared with the aripiprazole group (−2.25 versus −1.82, resp.; *P* = 0.047). The percentage of patients with treatment-emergent high prolactin at any time (10.7% versus 4.5%, resp.) or endpoint (4.9% versus 3.6%, resp.) was not significantly different between treatment groups.

Significant decreases in the pomaglumetad methionil treatment group compared with the aripiprazole treatment group were reported for BMI [standard error: SE] (−1.0 [0.1] versus 0.2 [0.2] kg/m^2^; *P* < 0.001) and waist circumference (SE) (−2.3 [0.3] versus 0.4 [0.6] cm; *P* < 0.001) (Table 4).

### 3.3. Efficacy Measures

There was a significant improvement within the pomaglumetad methionil and aripiprazole treatment groups in all the efficacy scores at Week 24 (Visit 12) when compared with baseline scores. However, the change in PANSS total scores for the aripiprazole treatment group was statistically significantly greater than the change for the pomaglumetad treatment group during the double-blind active treatment phase, as measured by the LS mean (SE) change from baseline (−15.58 [1.58] versus −12.03 [0.99]; *P* = 0.045) at Week 24 (Visit 12). Similarly, the change in positive symptoms (−4.62 [0.50] versus −3.40 [0.32]; *P* = 0.032) and general psychopathology symptoms (−7.85 [0.89] versus −5.80 [0.56]; *P* = 0.040) for the aripiprazole treatment group was statistically significantly greater than the change for the pomaglumetad treatment group. There were no significant differences reported between treatment groups in the improvement of negative symptoms as measured by the PANSS negative scale and the NSA-16 scale at Week 24 (Visit 12). No significant differences were reported between treatment groups at Week 24 (Visit 12) on the CGI-S scale (Table 5).

At the end of the double-blind active treatment phase, significantly more responders (defined as those having ≥30% decrease in PANSS total scores from baseline) were noted in the aripiprazole treatment group compared with the pomaglumetad methionil group (16.1% versus 9.1%; *P* = 0.017).

## 4. Discussion

Weight gain prevention has become a major research interest since drug-induced weight gain is a risk factor for diabetes and cardiovascular problems and is a significant cause of antipsychotic treatment noncompliance [30]. Aripiprazole has generally been considered to have a lower propensity for weight gain than some other antipsychotics [3] but may still be associated with weight increase in some populations, depending upon previous antipsychotic exposure [31, 32]. The significant separation of pomaglumetad methionil and aripiprazole on weight outcome offers the opportunity to demonstrate a unique weight attribute of pomaglumetad methionil. In the current study, the aripiprazole group showed a very small increase in weight from baseline (0.4 kg), consistent with previous reports. In contrast, the pomaglumetad methionil group showed significant weight loss from baseline (−2.8 kg), and significantly more pomaglumetad methionil-treated patients reported a ≥7% decrease in weight from baseline compared with aripiprazole-treated patients at endpoint and at any time during the double-blind study. It is not clear from the present results whether any of the reported weight loss was a consequence of the discontinuation of previous antipsychotic treatment, resulting in shedding of excess weight gained during that treatment, was due to anticraving effects of pomaglumetad methionil treatment, or both [33]. Regardless, the results suggest that the weight profile for pomaglumetad is unique compared with current antipsychotics.

Most AEs were reported at similar incidence rates in both treatment groups. However, nausea occurred more often in the pomaglumetad methionil group, and there was a significantly higher incidence of akathisia, dyspepsia, pyrexia, and nasal congestion in the aripiprazole treatment group. The higher incidence of akathisia in the aripiprazole group is consistent with previously reported outcomes for this compound [34, 35]. Nausea and vomiting have been observed in other pomaglumetad methionil trials, and aripiprazole has also been previously associated with gastrointestinal-related AEs. The observed decrease in weight for the pomaglumetad methionil group does not appear to be a consequence of nausea and vomiting because for most patients the gastrointestinal events occurred only during the first weeks of treatment, whereas the time course of weight changes was gradual and persistent across the 24 weeks. Suicidal ideation (40% to 50%) and behavior (20% to 50%) are frequent in patients with schizophrenia [36]. In the present study, rates of suicidal ideation and behavior were not significantly different between the treatment groups during the double-blind active treatment phase, with 1.4% of patients in the pomaglumetad methionil group exhibiting suicidal behavior.

There were no clinically relevant laboratory findings, and there were no clinically significant findings on vital signs or ECGs for the pomaglumetad methionil group compared with the aripiprazole group. Analysis of fasting lipids and glucose generally did not show significant treatment differences, although there were significantly fewer patients with a shift from normal/borderline triglycerides to high triglycerides at any time in the pomaglumetad methionil group. It is possible that a longer duration of observation may be needed to observe further differences in metabolic parameters as a result of the decreases in weight.

The overall greater number of SAEs and discontinuations due to AEs in the pomaglumetad methionil group appeared to be primarily driven by disease state-related AEs (psychosis and schizophrenia) and may be related to inferior efficacy compared with aripiprazole as well as manifestations of the underlying disease state.

The lack of placebo in the current study limits our interpretation of efficacy, so efficacy outcomes were secondary. However, the efficacy of pomaglumetad methionil was inferior compared with aripiprazole in this trial. Definitive placebo-controlled efficacy studies were being conducted in parallel to this study. The present study was stopped early when the pomaglumetad methionil schizophrenia monotherapy development program was stopped, based on lack of efficacy in an acute placebo-controlled efficacy study [17] as well as early stopping of a second acute trial due to futility [18]. This early stopping had minimal impact on the double-blind active treatment phase results of the present study, however, since enrollment was complete and 97% of patients had already completed or discontinued the double-blind active treatment phase of the study at the time of termination. (As noted in the Results Section, the most common reason for discontinuation during the open-label active treatment phase was sponsor decision (48.9%), reflecting early termination of the study.) Subgroups defined by clinical parameters, markers that may reflect an underlying hyperglutamatergic tone, or genotype are currently being explored across the completed pomaglumetad methionil studies to understand if there may be a more responsive subgroup of patients.

## 5. Conclusions

Treatment for 24 weeks with pomaglumetad methionil resulted in significantly less weight gain compared with aripiprazole. However, inferior efficacy in this longer term study along with the recent results of acute placebo-controlled pivotal efficacy studies suggests that pomaglumetad methionil is not effective in a broad schizophrenia patient population. More research is needed to determine whether a targeted patient population may be responsive to a glutamatergic treatment for the treatment of schizophrenia.

## Figures and Tables

**Table 1:** Patient demographics and baseline characteristics.

Variable	Double-blind treatment phase			
	Pomaglumetad methionil *N* = 511	Aripiprazole *N* = 161	Total *N* = 672	*P* value^a^
Sex				
Female, *n* (%)	185 (36.2)	55 (34.2)	240 (35.7)	0.706
Male, *n* (%)	326 (63.8)	106 (65.8)	432 (64.3)	
Age				
Mean, year (SD)	42.29 (10.86)	42.95 (10.95)	42.45 (10.88)	0.500
Range, year	18.4–64.4	21.3–65.0	18.4–65.0	
Ethnicity, *n* (%)				
Hispanic or Latino	73 (14.3)	23 (14.3)	96 (14.3)	>0.999
Not Hispanic or Latino	438 (85.7)	138 (85.7)	576 (85.7)	
Race, *n* (%)				
American Indian or Alaskan Native	6 (1.2)	0 (0.0)	6 (0.9)	0.130
Asian	3 (0.6)	0 (0.0)	3 (0.4)	
Black or African American	238 (46.6)	64 (39.8)	302 (44.9)	
Multiple	5 (1.0)	4 (2.5)	9 (1.3)	
White	259 (50.7)	93 (57.8)	352 (52.4)	
Weight				
Mean, kg (SD)	89.8 (22.04)	90.18 (22.80)	89.90 (22.21)	0.851
BMI				
Mean, kg/m^2^ (SD)	30.35 (7.32)	30.72 (7.63)	30.44 (7.39)	0.575
Waist circumference				
Mean, cm (SD)	100.90 (16.82)	101.80 (18.01)	101.11 (17.10)	0.559

^a^ *P* value is from Fisher's exact test for categorical data and is from a single-factor analysis of variance model (ANOVA) with fixed effect of treatment for continuous variable.

BMI: body mass index; cm: centimeter; *N*: total number of patients in each treatment group; *n*: number of patients in each category; kg: kilogram; kg/m^2^: kilogram per square meter; SD: standard deviation.

**Table 2:** Incidence of serious adverse events, discontinuations due to adverse events, and treatment-emergent adverse events during the double-blind treatment phase.

Safety measure	Double-blind treatment phase			*P* value^a^
	Pomaglumetad methionil *N* = 511	Aripiprazole *N* = 161	Total *N* = 672	
SAEs	42 (8.2)	5 (3.1)	47 (7.0)	0.032*
Discontinuations due to AEs	83 (16.2)	14 (8.7)	97 (14.4)	0.020*
TEAEs	370 (72.4)	109 (67.7)	479 (71.3)	0.272

*P < 0.05.

^a^ *P* values are from Fisher's exact test.

AE: adverse event; *N*: total number of patients in each treatment group; SAE: serious adverse event; TEAE: treatment-emergent adverse event.

**Table 3:** Incidence of most common treatment-emergent adverse events ≥3% in any treatment group and/or with statistically significant treatment difference, by preferred term.

MedDRA preferred term	Double-blind treatment phase			*P* value^a^
	Pomaglumetad methionil *N* = 511 *n* (%)	Aripiprazole *N* = 161 *n* (%)	Total *N* = 672 *n* (%)	
Nausea	98 (19.2)	18 (11.2)	116 (17.3)	0.023*
Insomnia	50 (9.8)	19 (11.8)	69 (10.3)	0.459
Headache	57 (11.2)	10 (6.2)	67 (10.0)	0.071
Vomiting	41 (8.0)	12 (7.5)	53 (7.9)	>0.999
Nasopharyngitis	38 (7.4)	8 (5.0)	46 (6.8)	0.371
Blood creatine phosphokinase increased	29 (5.7)	4 (2.5)	33 (4.9)	0.141
Anxiety	22 (4.3)	9 (5.6)	31 (4.6)	0.519
Decreased appetite	22 (4.3)	6 (3.7)	28 (4.2)	>0.999
Diarrhea	20 (3.9)	8 (5.0)	28 (4.2)	0.651
Schizophrenia	24 (4.7)	2 (1.2)	26 (3.9)	0.058
Dizziness	17 (3.3)	8 (5.0)	25 (3.7)	0.343
Akathisia	13 (2.5)	12 (7.5)	25 (3.7)	0.007*
Dry mouth	15 (2.9)	5 (3.1)	20 (3.0)	>0.999
Fatigue	14 (2.7)	6 (3.7)	20 (3.0)	0.594
Back pain	9 (1.8)	5 (3.1)	14 (2.1)	0.341
Dyspepsia	5 (1.0)	6 (3.7)	11 (1.6)	0.027*
Pyrexia	2 (0.4)	4 (2.5)	6 (0.9)	0.032*
Nasal congestion	1 (0.2)	3 (1.9)	4 (0.6)	0.045*

*P < 0.05.

^a^ *P* values are from Fisher's exact test.

*N*: total number of patients in each treatment group; MedDRA: Medical Dictionary for Regulatory Activities; TEAE: treatment-emergent adverse event.

**Table 4:** Least-squares mean change from baseline to Week 24 (Visit 12) in the safety measures.

Measure total score	Double-blind Treatment Phase					*P* value^a^
	Pomaglumetad methionil (*N* = 511)		Aripiprazole (*N* = 161)		LS mean difference between pomaglumetad methionil and aripiprazole (SE)
	Baseline mean (SD)	Δ to Week 24 (Visit 12) LS mean (SE)	Baseline mean (SD)	Δ to Week 24 (Visit 12) LS mean (SE)	
Weight (kg)	89.80 (22.04)	−2.8 (0.4)	90.18 (22.80)	0.4 (0.6)	−3.2 (0.7)	<0.001*
BMI (kg/m^2^)	30.35 (7.32)	−1.0 (0.1)	30.72 (7.63)	0.2 (0.2)	−1.1 (0.2)	<0.001*
Waist circumference (cm)	100.9 (16.8)	−2.3 (0.3)	101.8 (18.0)	0.4 (0.6)	−2.7 (0.6)	<0.001*
EPS						
BAS	0.1 (0.5)	0.00 (0.03)	0.1 (0.4)	−0.04 (0.04)	0.04 (0.04)	0.353
SAS	0.5 (1.7)	−0.17 (0.06)	0.4 (1.2)	−0.20 (0.08)	0.03 (0.09)	0.698
AIMS	0.3 (1.2)	−0.11 (0.05)	0.3 (1.1)	−0.10 (0.07)	−0.01 (0.07)	0.924

*P < 0.001.

^a^ *P* values are from type III tests of LS mean differences between treatments at each visit from MMRM.

Δ: change from baseline; LS mean: least-squares means; AIMS: Abnormal Involuntary Movement Scale; BAS: Barnes Akathisia Scale; cm: centimeter; EPS: extrapyramidal symptoms; kg: kilogram; kg/m^2^: kilogram per square meter; MMRM: mixed-effects model with repeated measures; *N*: number of patients; SAS: Simpson-Angus Scale; SD: standard deviations; SE: standard error.

**Table 5:** Least-squares mean change from baseline to Week 24 (Visit 12) in the efficacy measures.

Measure	Double-blind treatment phase					*P* value^a^
	Pomaglumetad methionil (*N* = 511)		Aripiprazole (*N* = 161)		LS mean difference between pomaglumetad methionil and aripiprazole (SE)
	Baseline mean (SD)	Δ to Week 24 (Visit 12) LS mean (SE)	Baseline mean (SD)	Δ to Week 24 (Visit 12) LS mean (SE)	
PANSS						
Total score	77.9 (24.15)	−12.03 (0.99)	79.5 (22.19)	−15.58 (1.58)	3.55 (1.77)	0.045*
Positive	19.1 (6.75)	−3.40 (0.32)	19.5 (6.31)	−4.62 (0.50)	1.21 (0.56)	0.032*
Negative	20.4 (7.13)	−2.98 (0.31)	21.2 (7.10)	−3.34 (0.48)	0.36 (0.54)	0.509
General psychopathology	38.4 (13.08)	−5.80 (0.56)	38.7 (12.10)	−7.85 (0.89)	2.05 (1.00)	0.040*
CGI-S score	4.1 (0.79)	−0.51 (0.05)	4.1 (0.74)	−0.69 (0.08)	0.17 (0.09)	0.055
NSA-16 total score	46.3 (13.34)	−6.22 (0.68)	47.5 (13.48)	−6.37 (1.03)	0.15 (1.11)	0.891

*P < 0.05.

^a^ *P* values are from type III tests of LS mean differences between treatments at each visit from MMRM.

Δ: change from baseline; LS mean: least-squares mean; *N*: number of patients; CGI-S: Clinical Global Impression-Severity; MMRM: mixed-effects model with repeated measures; NSA-16: 16-Item Negative Symptom Assessment; PANSS: Positive and Negative Syndrome Scale; SD: standard deviations; SE: standard error.
